# Nonradiographic axial spondyloarthritis: expanding the spectrum of an old disease: A narrative review

**DOI:** 10.1097/MD.0000000000029063

**Published:** 2022-04-15

**Authors:** Marina Magrey, Sergio Schwartzman, Natasha de Peyrecave, Victor S. Sloan, Jeffrey L. Stark

**Affiliations:** a Case Western Reserve University School of Medicine at MetroHealth Medical Center, Department of Medicine, Division of Rheumatology, Cleveland, OH; b University Hospitals Cleveland Medical Center School of Medicine, Division of Rheumatology, Cleveland, OH; c Weill Cornell Medical College, Division of Rheumatology, New York, NY; d UCB Pharma, Brussels, Belgium; e Sheng Consulting LLC, Flemington, NJ; f Rutgers Robert Wood Johnson Medical School, Division of Rheumatology and Connective Tissue Research, New Brunswick, NJ; g The Peace Corps, Washington, DC; h UCB Pharma, Smyrna, GA.

**Keywords:** ankylosing spondylitis, disease burden, internists, nonradiographic axial spondyloarthritis, targeted therapies

## Abstract

Nonradiographic axial spondyloarthritis (nr-axSpA) represents a distinct phenotype within the spectrum of axial spondyloarthritis (axSpA), which is characterized by a range of clinical manifestations. Despite a high disease burden that is comparable to ankylosing spondylitis (also known as radiographic axSpA), there is an unmet need to recognize and effectively manage patients with active nr-axSpA.

A targeted literature search was conducted in OVID (MEDLINE and Embase databases) to identify articles on nr-axSpA, including its definition, demographics, epidemiology, burden, diagnosis, clinical presentation, and treatment guidelines.

The lack of adequate epidemiological data and incomplete understanding of nr-axSpA among rheumatologists and nonrheumatologists contributes to delayed referrals and diagnosis. This delay results in a substantial burden on patients, physically and psychologically, and the healthcare system. Targeted therapies, such as biologics, including inhibitors of tumor necrosis factor or interleukin-17A, have been approved and utilized for the management of nr-axSpA, and other novel therapeutics with different mechanisms of action are in development. Raising awareness among US internists regarding the prevalence of nr-axSpA, disease burden, clinical presentation, diagnostic tools, and available treatments is important for improved disease management.

Future clinical investigations focusing on the development of markers that aid early diagnosis and predict treatment response may also improve the management of nr-axSpA. This review provides an overview of nr-axSpA with the aim of raising awareness of the disease among US internists, with an overarching goal to contribute toward the improved recognition and timely referral of these patients to rheumatologists for diagnosis and management.

## 1 Introduction

### 1.1 The spondyloarthritides (SpA) family

Spondyloarthritis (SpA; formerly seronegative spondyloarthritis or spondyloarthropathy) encompasses diseases sharing common features, including an association with major histocompatibility complex class I antigen human leukocyte antigen B27 (HLA-B27) allele, axial involvement (inflammation of sacroiliac joints [SIJs] and spine resulting in chronic back pain [CBP]), peripheral joint arthritis, enthesitis, dactylitis, and extra-articular manifestations (recently termed extramusculoskeletal manifestations), including inflammatory bowel disease (IBD), psoriasis, and acute anterior uveitis.^[1–4]^ SpA can be broadly categorized into axial SpA (axSpA) and peripheral SpA. AxSpA is a chronic inflammatory rheumatic disease with predominant involvement of the axial skeleton. Patients with axSpA can be further classified into ankylosing spondylitis (AS; also referred to as radiographic axSpA; presence of definite radiographic sacroiliitis above a specific threshold of detection through radiographs) and nonradiographic axSpA (nr-axSpA; absence of definite radiographic sacroiliitis above the threshold).^[1–3]^

### 1.2 Evolution of axSpA

**Figure 1:**
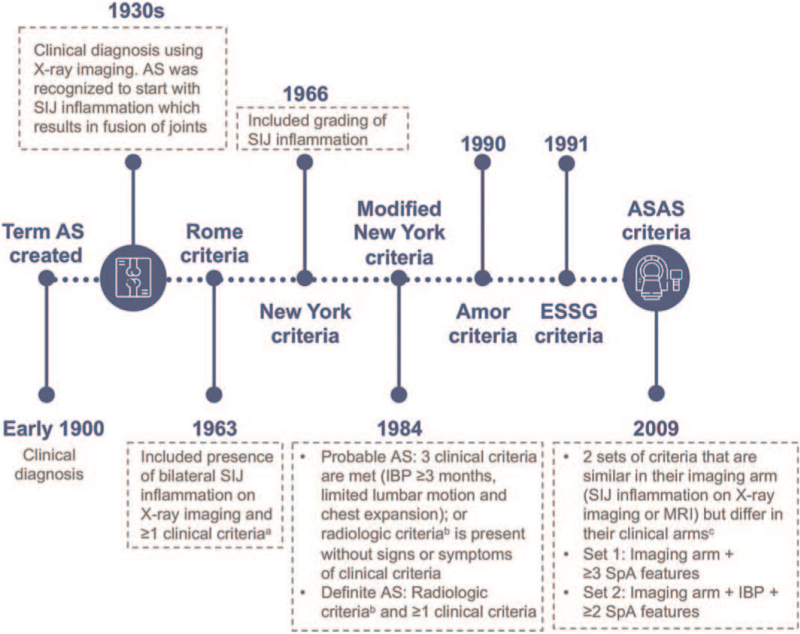
Evolution of axSpA classification criteria. In the early 1900s, the term “AS” was created to describe the condition where there was inflammation in the spine and stiffness of all or part of the spine.^[5]^ The use of X-ray imaging in the 1930s,^[6]^ contributed to the creation of the 1963 Rome classification criteria of axSpA, 1966 New York classification criteria and then the 1984 modified New York classification criteria (where AS was probable or definitive depending on whether radiologic criteria was present and on the number of clinical criteria present).^[7]^ Subsequently, the Amor classification criteria was introduced in 1990 and the ESSG classification criteria in 1991, preceding the ASAS classification criteria for axSpA in 2009, widening the scope of axSpA to include both AS and nr-axSpA.^[1,2]^^a^Clinical criteria include: low back pain and stiffness >3 months; pain and stiffness in the thoracic region; limited lumbar movement; limited chest expansion; history or evidence of iritis or its sequelae; ^b^Sacroiliitis grade ≥2 bilaterally or sacroiliitis grade 3–4 unilaterally; ^c^Clinical arm includes the following SpA features: IBP, arthritis, enthesitis (heel), anterior uveitis, dactylitis, psoriasis, inflammatory bowel disease (Crohn disease/ulcerative colitis), good response to NSAIDs, family history of SpA, HLA-B27 positivity, and elevated CRP or ESR levels. AS = ankylosing spondylitis, ASAS = Assessment of Spondyloarthritis International Society, axSpA = axial spondyloarthritis, CRP = C-reactive protein, ESR = erythrocyte sedimentation rate, ESSG = European Spondyloarthopathy Study Group, HLA-B27 = human leukocyte antigen-B27, IBP = inflammatory back pain, MRI = magnetic resonance imaging, nr-axSpA = nonradiographic axial spondyloarthritis, NSAID = nonsteroidal antiinflammatory drug, SIJ = sacroiliac joint, SpA = spondyloarthritis.

In 1893, Vladimir Mikhailovich Bechterew recognized a chronic inflammatory condition of the spine, resulting in spinal stiffness. In 1904, Eugene Frankel coined the term AS. Subsequently, AS was recognized to begin with SIJ inflammation progressing to the spine, resulting in fusion of part/all of the axial skeleton.^[5,6]^ AS classification criteria were first promulgated at the 1963 Rome conference and then modified to the 1966 New York classification criteria, and eventually the 1984 modified New York classification criteria (Fig. 1).^[7]^ Syndesmophytes were considered a hallmark of AS.^[6]^

Around the early 1980s, family studies of HLA-B27-positive AS probands showed an increased prevalence of chronic inflammatory back pain (IBP), and thoracic pain and stiffness among first-degree relatives without radiologically detectable sacroiliitis.^[8,9]^ The findings suggested that the disease was broader than specified by the Rome or (modified) New York classification criteria, and includes individuals with AS symptoms but without radiologically detectable SIJs or spinal abnormalities.^[8]^ nr-axSpA thus existed without methods to classify it.

Increased disease awareness has led to the development of the 1990 Amor classification criteria, the 1991 European Spondyloarthopathy Study Group (ESSG) classification criteria, and the 2009 Assessment of Spondyloarthritis International Society (ASAS) classification criteria for axSpA, widening the scope of axSpA to include AS and nr-axSpA (Fig. 1).^[2,3]^ According to ASAS classification criteria, patients with CBP for at least 3 months and an age of onset before 45 years were considered to have axSpA if they displayed sacroiliitis on radiographs or magnetic resonance imaging (MRI) plus at least 1 other SpA feature; alternatively, patients can have HLA-B27 positivity plus at least 2 other SpA features.^[2,3]^

Recently, nr-axSpA was indexed to an International Statistical Classification of Diseases and Related Health Problems-10 diagnostic code distinct from the code for AS, allowing accurate and precise identification of patients with nr-axSpA in databases for clinical/billing purposes.^[10]^ While this is a step forward, there remains a need to educate US internists regarding disease manifestations, severity, the natural course of disease and treatment options. This narrative review summarizes evidence from a targeted literature search on nr-axSpA epidemiology and burden, the challenges around diagnosing patients with nr-axSpA, and treatment recommendations. This pragmatic approach to providing an overview of nr-axSpA aims to raise awareness of the disease among US internists, with an overarching goal to contribute toward the improved recognition and timely referral of these patients to rheumatologists for diagnosis and management.

## 2 Methods

A targeted literature search was conducted to identify articles which provided information relevant for the aim of this review (see section 2.1 for details). Articles published in peer-reviewed scientific journals were included. Articles were excluded if they were not written in the English language or not published in peer-reviewed scientific journals.

### 2.1 Search strategy

Bibliographic research was conducted in OVID, using the MEDLINE and Embase databases, using the following keywords or combination of keywords (“CORRONA” OR “GESPIC” OR “DESIR” OR (“SPACE” AND “database”)) AND ((“non-radiographic” AND (“axSpA” OR “axial spondyloarthritis”)) OR (“axSpA” OR “axial spondyloarthritis” OR “AS” OR “ankylosing spondylitis”)). Articles were included if they were published between January 2004 and November 2019; or before 2004 if they were considered to provide information on the historical background of axSpA. Selected articles, published between December 2019 and November 2021, were additionally included based on the authors’ expertise and knowledge of the evolving literature. Articles written in the English language and published in peer-reviewed scientific journals were further screened. Case reports were excluded.

### 2.2 Study selection

Relevant articles were identified based on the title and abstract. Articles that provided information on the demographics and epidemiology of nr-axSpA, clinical presentation and burden of disease, the evolution of axSpA and definition of disease, diagnosis and referral strategies, and treatment guidelines, were included. Nine hundred and thirty-five articles were included from the initial search, and 70 articles from the search as well as 11 articles selected by the authors were included and discussed in the present review. This review does not need ethical approval as no human or patient data were utilized.

## 3 Results and discussion

### 3.1 Demographics and epidemiology of nr-axSpA

Epidemiological information on nr-axSpA is limited in the US owing to the lack of diagnosis/billing codes for nr-axSpA until recently, thus restricting the identification of patients.^[11]^ The evolution of classification criteria, differences in data collection methods, and study design contribute to variability in prevalence data. Epidemiological data suggest a higher prevalence than that reported in clinical studies, likely due to lower rates of diagnosis in the community.^[12]^

Using ASAS classification criteria, 1 US-based study estimated the mean prevalence of axSpA to be 0.70% between 1985 and 2011, whereby nr-axSpA represented 0.35% and AS the other 0.35%.^[13]^ These data are lower than those reported in the US 2009 to 2010 National Health and Nutrition Examination Survey (axSpA: 1.0%–1.4%; AS: 0.52%–0.55%) that used Amor and ESSG classification criteria (which do not consider MRI findings for classification). At the time, the requirement for MRI of the SIJ and/or HLA-B27 typing made ASAS classification criteria, which are more stringent than Amor or ESSG classification criteria, unfeasible for population studies.^[14]^ Prevalence rates might differ if the study used ASAS classification criteria; data may thus inaccurately represent the true prevalence of axSpA.

In the Consortium of Rheumatology Researchers of North America (CORRONA) psoriatic arthritis/SpA registry, involving 407 patients fulfilling ASAS classification criteria, the female prevalence was higher in nr-axSpA (43%) than in AS (34%).^[15]^ The prevalence of axial SpA (PROSpA) study supported these data, wherein 54% of patients with nr-axSpA were female, compared with 43% of patients with AS.^[16]^ This pattern is consistent with observations in the French Outcome of Recent Onset Spondyloarthritis (DESIR) and German Spondyloarthritis Inception Cohort.^[17,18]^ Data in the US show that the mean age of onset ranges from 20 to 29 years and is similar between nr-axSpA and AS cohorts.^[16]^ Since disease onset occurs when women might consider pregnancy, it is important to ensure optimal management of axSpA in women.

### 3.2 nr-axSpA: a phenotype within a disease spectrum

Progression from nr-axSpA to AS is defined by the development of definite radiographic sacroiliitis of the SIJ on plain pelvic radiographs, based on the modified New York classification criteria.^[1–3]^ Few longitudinal studies have assessed the likelihood of nr-axSpA progressing to AS. Based on studies in the US, Europe and China, 1% to 60% of patients with nr-axSpA could take 2 to 15 years to progress to AS^[19–21]^; approximately 30% of patients with nr-axSpA may never progress to AS despite IBP or elevated C-reactive protein (CRP)/erythrocyte sedimentation rate (ESR) levels.^[22]^ Comparisons among these studies may be limited as they include patients from different parts of the axSpA spectrum. Additionally, more than 40% of patients may be omitted from analyses because of loss to follow-up.^[20]^ Nevertheless, these data suggest that the classification criteria used in these studies identified patients whose condition was unlikely to progress to AS, or that nr-axSpA-to-AS progression may take more than 15 years in some patients. Studies have identified modifiable predictors of radiographic progression (including smoking and objective inflammation) and nonmodifiable ones (Table 1).^[23,24]^

**Table 1:**
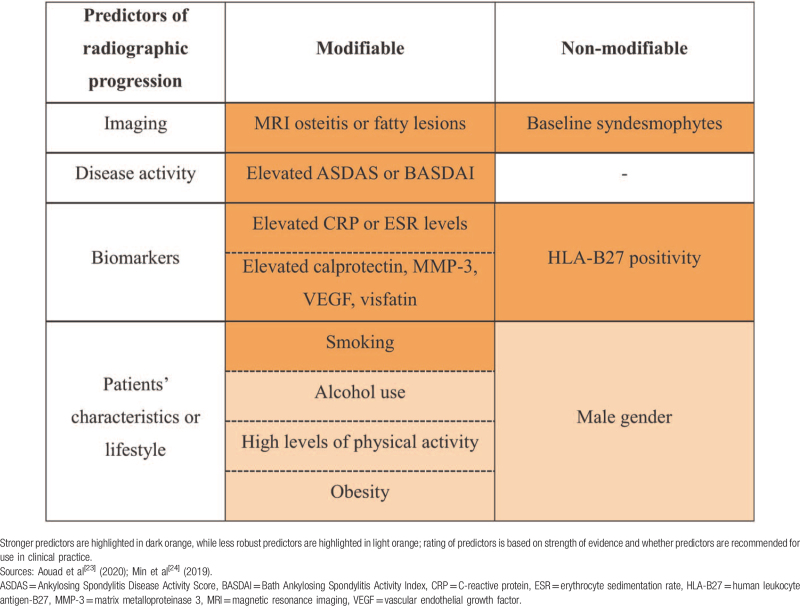
Evidence-based predictors of radiographic progression in axial spondyloarthritis.

Thus, axSpA is a paradigm of potential progressive structural damage, whereby nr-axSpA and AS represent 2 phenotypes within the axSpA spectrum. One can use rheumatoid arthritis as an analogy: rheumatoid arthritis is viewed as a disease spectrum with erosive and nonerosive disease.^[25]^ nr-axSpA is therefore a new name for an old disease.

### 3.3 Gender differential in clinical presentation

Traditionally, based on patterns in AS, nr-axSpA was initially considered to predominantly affect males.^[12,26]^ With the evolution of the concept of nr-axSpA, it has been recognized that nr-axSpA is equally prevalent in females.^[26]^ Some differences have been observed between genders in the clinical presentation of axSpA: Females tend to have less structural damage than males, and may be less likely to progress from nr-axSpA to AS.^[17,26]^ Females generally have greater peripheral involvement (dactylitis and enthesitis), neck involvement, and widespread pain compared with males, while males generally have more structural damage in SIJs/spine (resulting in decreased spinal mobility and ribcage flexibility).^[26]^ Disease manifestations including IBD and psoriasis are generally more common in females than males, while acute anterior uveitis is generally more common in males.^[26]^

### 3.4 Burden of disease

Despite no definitive structural damage in patients with nr-axSpA, studies have shown a comparable disease burden between nr-axSpA and AS. An analysis of the CORRONA registry showed that although patients with nr-axSpA were younger and had shorter symptom duration than patients with AS, active disease status (Bath Ankylosing Spondylitis Disease Activity Index, Ankylosing Spondylitis Disease Activity Score) and functional disability (Bath Ankylosing Spondylitis Functional Index) scores were similar between the 2 groups.^[15]^ Consistent observations were made in patients considered to be tumor necrosis factor inhibitor (TNFi)-naïve; both subgroups had comparable disease activity, pain, fatigue, and health-related quality of life (QoL).^[17,27]^ One study showed that patients with nr-axSpA had significantly higher disease activity than AS (Bath Ankylosing Spondylitis Disease Activity Index 4.1 vs 2.7), and lower QoL (ASQoL Questionnaire Score of 8.8 vs 6.4).^[28]^

Comorbidities contribute to the burden of nr-axSpA. The ASAS-COMOrbidities in SPondyloArthritis study showed that globally, common comorbidities among patients with SpA included hypertension (22.4%), osteoporosis (13%) and gastroduodenal ulcer (11%).^[29,30]^ Hip arthritis is also common, with a prevalence of 9% among patients with nr-axSpA (AS: 19%–36%), and can be associated with increased disability, and decreased QoL and employability.^[31,32]^ Hip involvement is more prevalent in patients with a younger age of disease onset; these patients may experience the burden of complications related to total hip replacements.^[32]^ The prevalence of fibromyalgia is 20.3% among patients with nr-axSpA (AS: 13.8%).^[33]^ Patients with nr-axSpA/AS and fibromyalgia have significantly worse disease activity, function, fatigue and QoL, and suffer work impairments.^[34]^ The overall impact of fibromyalgia, based on Fibromyalgia Impact Questionnaire-physical impairment and Fibromyalgia Impact Questionnaire-total score, was significantly higher in males than females.^[35]^ A systematic review and meta-analysis showed a similar prevalence of depression between nr-axSpA (36%) and AS (38%). Pooled data of both subgroups showed that those with depression had significantly worse disease activity.^[36]^ Furthermore, axSpA can substantially impact patients and society financially. The CORRONA study showed that nr-axSpA/AS subgroups experienced comparable work productivity loss, but that presenteeism and overall activity impairment were significantly greater among patients with nr-axSpA than in those with AS.^[15]^

### 3.5 Diagnosis and referral strategies

**Figure 2:**
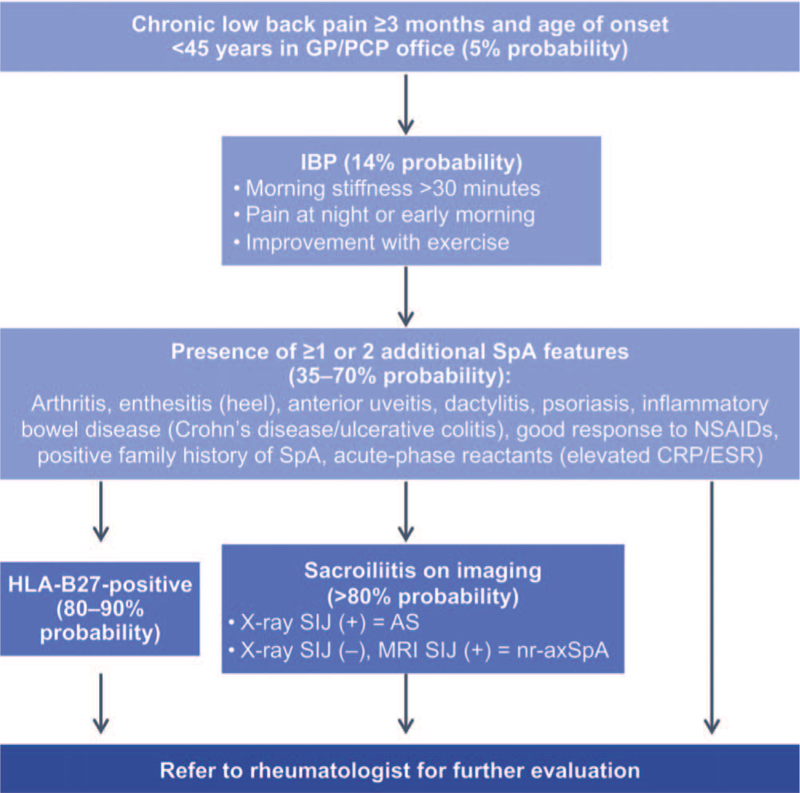
Referral strategy and clinical diagnosis of axSpA. The diagnosis of axSpA can be challenging and is highly dependent on clinical experience and intuition of the treating physician.^[38]^ The first step involves evaluating a patient with chronic back pain to determine whether the back pain is inflammatory or mechanical in nature.^[38]^ IBP is characterized by morning stiffness for >30 min, pain at night or early morning, and improves with exercise but does not improve with rest.^[2,12]^ For those with IBP, clinicians next evaluate the patient for the presence of SpA features. HLA-B27 testing may be performed, because a positive HLA-B27 test result is associated with an increased likelihood that the patient has axSpA.^[2]^ Imaging is also important for the recognition and diagnosis of axSpA. Patients with AS will have structural damage of the SIJ that is evident on X-ray. Patients with nr-axSpA will not have sacroiliitis evident on X-ray, but may have evidence of sacroiliitis by MRI.^[2,3]^ For those with chronic back pain for at least 3 months, an age of onset before 45 years and features of SpA, the presence of objective signs of inflammation (such as elevated CRP and evidence of sacroiliitis by MRI) can help improve the confidence of an axSpA diagnosis. This figure was adapted from Rudwaleit et al^[38]^ (2004). AS = ankylosing spondylitis, axSpA = axial spondyloarthritis, CRP = C-reactive protein, ESR = erythrocyte sedimentation rate, GP = general practitioner, HLA-B27 = human leukocyte antigen-B27, IBP = inflammatory back pain, MRI = magnetic resonance imaging, nr-axSpA = nonradiographic axial spondyloarthritis, NSAID = nonsteroidal anti-inflammatory drug, PCP = primary care practitioner, SIJ = sacroiliac joint, SpA = spondyloarthritis.

The diagnosis of nr-axSpA remains challenging with no available diagnostic criteria.^[12,37,38]^ A survey of 1690 US physicians revealed that long wait times and insurance restrictions were some barriers to early referral of patients with suspected axSpA.^[39]^ Accurate referral strategies can guide initial evaluation for suspected axSpA.^[12]^ IBP is a frequently used referral criterion. However, not all patients with axSpA have IBP and in a typical axSpA cohort, 63% to 92% of patients have IBP based on various classification criteria.^[21]^ While a common difficulty among providers is in identifying features suggestive of IBP,^[39]^ when IBP is considered alongside at least 1 other SpA feature, this can help enable appropriate referrals.^[40]^ ASAS proposed an early referral strategy, with the aim of maximising sensitivity; patients with CBP for at least 3 months and an age of onset before 45 years should be referred to a rheumatologist if IBP or at least 1 SpA feature is present (Fig. 2).^[12,41]^ A retrospective comparison of 13 referral strategies showed that while the ASAS strategy was the most effective at ensuring that no patients with axSpA were missed (high sensitivity), 1 caveat was that it identified patients who did not have axSpA (low specificity).^[42]^ Although the optimum referral strategy may depend on the healthcare environment, axSpA should be considered in patients with CBP and patients with suspected axSpA should be referred to a rheumatologist.^[12]^

#### 3.5.1 SpA features: imperfect predictors but useful for screening

While SpA features are not perfectly predictive of SpA, they can be informative when screening patients. One example is a positive family history (PFH) of SpA, described as a family history of AS, IBD, psoriasis, acute uveitis, or reactive arthritis in first- or second-degree relatives.^[2]^ US axSpA diagnosis data show that 24% of patients with nr-axSpA (AS: 18%) have a PFH of SpA.^[16]^ Another example is HLA-B27 positivity, which has been associated with early disease onset and disease progression.^[43]^ The US age-adjusted prevalence of HLA-B27 is 6.1%, but is lower in African-Americans (1.1%) and Hispanics (4.6%).^[14]^ The absence of HLA-B27 positivity does not rule out a diagnosis.^[44]^ CRP and ESR levels should also be considered.^[45]^ Globally, lower CRP and ESR levels are seen in patients with nr-axSpA than AS.^[17,27,46]^ However, CRP levels are normal in 40% to 61% of patients with axSpA, implying that CRP levels are nonspecific for active disease.^[45,47]^ CRP levels can also be influenced by body mass index, obesity, and treatment.^[47,48]^ Therefore, it is important to have as much information as possible about a patient when referring them to a rheumatologist.

#### 3.5.2 Imaging

**Figure 3:**
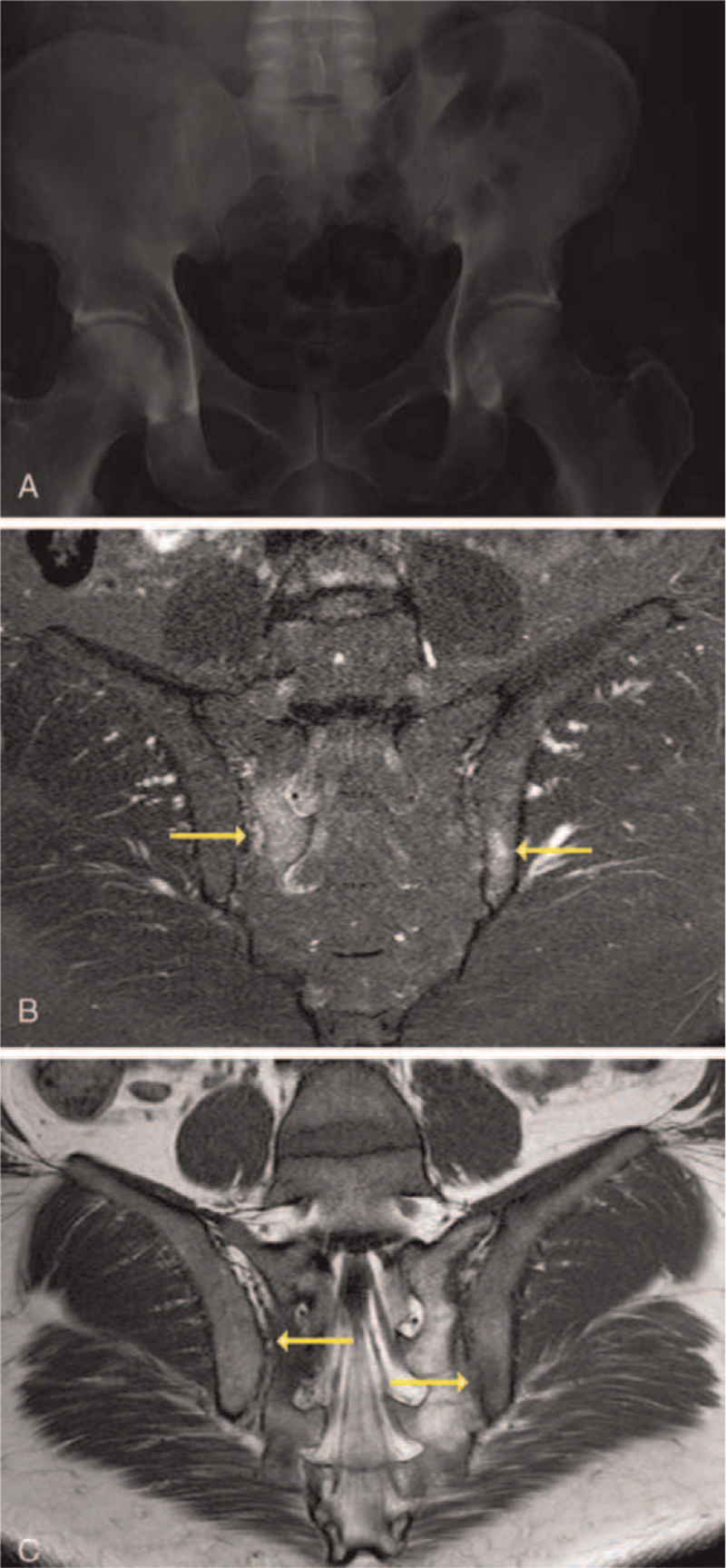
MRI plays an important role in the diagnosis of nr-axSpA. Imaging plays an important role in the diagnosis of nr-axSpA. In patients with nr-axSpA, X-ray images may be completely normal as these images do not allow detection of early signs of axial inflammation.^[49]^ The use of MRI of SIJs enables detection of osteitis (undetectable by X-ray) before radiographic sacroiliitis appears.^[49–51]^ (A) Shown is an X-ray image of a pelvis in a 45-year-old African-American patient with a 3-month history of low back and ankle pain. Pain wakes him at night and gets worse with prolonged sitting. The patient is HLA-B27-negative with a CRP level of 8.6 mg/dL. (B) Shown is an image of a STIR image showing high intensity edema (yellow arrows) on the sacral side of the right SIJ and iliac side of the left SIJ in the same patient. (C) Shown is a T1-weighted image with hypointensity areas showing edema with erosion (yellow arrows) on the sacral side of the right SIJ and iliac side of the left SIJ in the same patient. Patient images were provided courtesy of Dr Marina Magrey. axSpA = axial spondyloarthritis, CRP = C-reactive protein, MRI = magnetic resonance imaging, nr-axSpA = nonradiographic axial spondyloarthritis, SIJ = sacroiliac joint, STIR = short tau inversion recovery.

Imaging plays a pivotal role in axSpA diagnosis. Although abnormal pelvic X-rays are a quintessential part of diagnosing AS, plain radiography is typically normal in nr-axSpA. Plain radiography allows visualization of structural consequences of inflammation, but does not allow the detection of axial inflammation itself (Fig. 3A).^[49]^ This has led to the use of MRI of SIJs (fat-suppressed short tau inversion recovery or fat-enhanced T1-weighted sequences), which enables detection of osteitis (undetectable by X-ray) and structural lesions (erosions and fat metaplasia), before radiographic sacroiliitis appears (Fig. 3B).^[49–51]^ In patients with normal/equivocal X-rays, MRI of SIJs should thus be performed if axSpA is suspected.^[12]^

The specificity of MRI findings depends on the size, intensity, and location of osteitis, which must be periarticular, subchondral, and visible on at least 2 slices.^[51]^ MRI of SIJs should not be used alone to characterize nr-axSpA. MRI scans of healthy individuals (23%) and postpartum women (57%) met the ASAS classification criteria definition of a “positive MRI”.^[52]^ Inflammatory SIJ lesions were also observed in MRI scans of athletes, cases of trauma and degenerative SIJ arthritis.^[53,54]^ Using MRI alone can therefore result in an incorrect diagnosis of nr-axSpA. Additionally, a lack of formal training of rheumatologists and radiologists can result in different interpretations of diagnostic images, leading to misdiagnoses.^[37]^ Recent consensus recommendations for acquiring and interpreting MRI in the diagnosis of axSpA should help standardize practice and facilitate a more consistent as well as reliable approach to diagnosis.^[55]^ This would then avoid the inevitable false-positive and false-negative inference of axSpA from MRI.^[56]^ Nevertheless, MRI remains important, and modalities are being developed with improved sensitivity in detecting erosions and other chronic SIJ lesions.^[57]^

Bone scintigraphy is a screening tool that can detect axSpA-related sacroiliitis, specifically by identifying regions of inflammation and high bone turnover within SIJs.^[58]^ Although this technique has been used in the detection of acute sacroiliitis for the early diagnosis of axSpA,^[57]^ its overall sensitivity is low (around 50%) relative to MRI (around 82%),^[59–61]^ exposes patients to higher levels of radiation relative to MRI, and has not been recommended for the diagnosis of sacroiliitis as part of axSpA.^[49]^ MRI scans of SIJs have therefore evolved as the mainstay of diagnostic imaging in nr-axSpA, allowing early diagnosis of axSpA.^[50]^

#### 3.5.3 Missed or incorrect diagnosis

A sizeable patient population has a missed or incorrect diagnosis, and therefore, delayed diagnosis. The relatively high sensitivity of IBP as an indicator for axSpA (75%) makes it useful for screening at-risk patients,^[38]^ however a diagnosis would be missed in one-quarter of patients if screening depended on IBP alone.^[12]^

Nonrheumatologists are often the first to see patients with axSpA and may be unfamiliar with differentiating axSpA from other causes of back pain. A retrospective study from 2000 to 2012 showed that 37% of patients with AS were diagnosed by rheumatologists, while the remaining diagnosed by primary care physicians (26%), chiropractors/physical therapists (7%), orthopedic surgeons (4%), pain clinics (4%), in acute care (3%) and other settings (19%).^[62]^ Patients may seek initial care from specialists, such as a dermatologist to treat psoriasis; some may choose alternative/complementary medicine. Patients may also seek routine care from chiropractors, particularly in rural areas, where access to medical specialists may be limited.^[37]^ The PROSpA study showed that even US rheumatologists missed axSpA diagnosis in 40% of patients, and diagnosis was delayed by 14 years on average.^[37]^ These data suggest a need to raise awareness among internists regarding axSpA signs and symptoms.

### 3.6 Treatment

#### 3.6.1 Recommendations for management of nr-axSpA

There are several recommendations for managing nr-axSpA.^[49,63,64]^ Physical therapy along with nonsteroidal anti-inflammatory drugs (NSAIDs), including cyclooxygenase-2 and noncyclooxygenase-2 inhibitors, is strongly recommended as first-line treatment, with NSAIDs prescribed at the maximum tolerated dose, assessing risk versus benefit.^[50,64]^

Patients who are intolerant or do not respond to at least 2 NSAIDs and still have active disease should be prescribed biologic disease-modifying antirheumatic drugs (bDMARDs), including TNFis and interleukin-17A (IL-17Ai).^[50,64]^

Treatment options that are not recommended for axial disease include conventional synthetic antirheumatic drugs (including methotrexate and sulfasalazine) and systemic glucocorticoids. Sulfasalazine and local injectable glucocorticoids are effective for managing peripheral manifestations, but when used alone, they are rarely/not effective for axial disease and do not modify disease progression.^[50,63]^

#### 3.6.2 Available evidence for treatment options

##### 3.6.2.1 Physical therapy

Physical therapy is strongly recommended as first-line treatment. The American College of Rheumatology, in partnership with the Spondyloarthritis Research and Treatment Network, and Spondylitis Association of America (ACR/SPARTAN/SAA) conditionally recommends active interventions (supervised exercise) over passive ones (massage, ultrasound, heat), and land-based exercises over aquatic interventions.^[63]^ Physical therapy interventions improve functioning and are beneficial to patients when used with pharmacologic treatments.^[65]^ Patients with nr-axSpA who underwent a 6-month intensive exercise program had significantly improved spinal mobility and disease activity (Ankylosing Spondylitis Disease Activity Score) versus those who did not exercise. Similar efficacy was observed in patients with nr-axSpA and AS.^[66]^

##### 3.6.2.2 NSAIDs and glucocorticoids

NSAIDs improve axSpA symptoms and are effective in patients with nr-axSpA.^[63,64]^ ACR/SPARTAN/SAA conditionally recommend continuous over on-demand NSAID treatment, and do not recommend any preferred NSAIDs. In patients who fail NSAIDs, ACR/SPARTAN/SAA strongly recommend against treatment with systemic glucocorticoids for axial disease. Local glucocorticoids can be used in patients with peripheral symptoms.^[63]^

In TNFi-naïve patients with active nr-axSpA, continuous NSAID use improved pain and function, with no observed difference between patients who received low NSAID doses before the study versus NSAID-naïve patients, or between continuous NSAID use versus reduced doses.^[67]^ A randomized clinical trial (RCT) of patients with axSpA also showed that the protective effect of NSAIDs against structural progression was specific to the NSAID.^[68]^ The combined use of an NSAID and a TNFi demonstrated higher clinical remission rates in NSAID-naïve patients with early axSpA versus NSAIDs only.^[69]^ Some patients do not respond to NSAIDs/NSAID-based regimens and with chronic use, experience side effects or lose response to treatment.^[64]^

##### 3.6.2.3 Biologic disease-modifying antirheumatic drugs

bDMARDs have transformed the nr-axSpA treatment paradigm.^[50]^ Patients with nr-axSpA whose disease activity remains high, despite treatment with at least 2 different NSAIDs at maximal doses for at least 4 weeks, should be treated with bDMARDS.^[63,64]^ ACR/SPARTAN/SAA strongly recommend the use of TNFi for nr-axSpA treatment based on efficacy and safety evidence from several clinical trials.^[63]^

RCTs have shown the efficacy of bDMARDs in patients with active axSpA with objective signs of inflammation (OSI). In patients who failed NSAIDs, RCTs demonstrated the effectiveness of the TNFis: adalimumab,^[70]^ etanercept,^[71]^ golimumab,^[72]^ certolizumab pegol (CZP)^[73–75]^; and the IL-17Ai: ixekizumab.^[76]^ In patients who were TNFi-naïve or failed TNFis, an RCT demonstrated the efficacy of the IL-17Ai: secukinumab.^[77]^

CZP is the only TNFi approved for treating nr-axSpA in the US.^[78]^ The RAPID-axSpA study first demonstrated CZP efficacy and safety in patients with nr-axSpA, along with improvements in QoL and patient-reported outcomes.^[73]^ SIJ and spinal MRI remission was achieved by nearly half of all patients with nr-axSpA at Week 12, and after 4 years of CZP treatment, there were limited changes in structural SIJ and spinal damage on X-ray and minimal net progression from nr-axSpA to AS.^[74]^ The 52-week placebo-controlled C-axSpAnd study further demonstrated the efficacy and safety of CZP plus nonbiologic background medication (NBBM) in patients with nr-axSpA, where it showed that adding CZP to NBBM was superior to placebo in improving symptoms and QoL.^[75]^ CZP also has negligible-to-low placental transfer and women of childbearing potential with nr-axSpA could benefit from CZP treatment.^[79]^

Ixekizumab and secukinumab are IL-17Ais approved for treating nr-axSpA in the US.^[80,81]^ The COAST-X study showed that in biologic-naïve patients with nr-axSpA who failed NSAIDs, ixekizumab resulted in significant improvements over placebo in symptoms and functions along with improvement in QoL, with no serious safety concerns.^[76]^ Similar clinical outcomes were seen in the PREVENT study, which demonstrated significant improvements with secukinumab over placebo in patients with nr-axSpA who were TNFi-naïve or failed TNFis, with no new safety findings.^[77]^

#### 3.6.3 Emerging therapies: IL-17 and IL-12/23 inhibitors

Other IL-17is such as bimekizumab (selectively inhibits IL-17A and IL-17F) and Janus kinase inhibitors such as upadacitinib, both with ongoing phase 3 trials, may be available in the future to treat patients with active nr-axSpA. IL-12/23 inhibitors (IL-12/23i) have also been tested in patients with nr-axSpA. Ustekinumab failed to demonstrate efficacy in phase 3 trials, while tildrakizumab is being studied in phase 2/3 trials.

#### 3.6.4 Future investigations

Important questions remain: What is the true rate of nr-axSpA to AS progression? How can these patients be identified efficiently? Which imaging modalities better evaluate such progression and what is the optimal timing interval? How can the effect of biologic therapies on radiographic progression be best evaluated? The effect of targeted therapy on co-manifestations/comorbidities of nr-axSpA needs to be better understood, and if treatment is inadequate, different strategies should be developed.

Identifying patients most likely to respond to specific therapies is important to improve disease management and inform the use of resource-intensive care in high-risk patients. No biomarker has consistently defined the disease or predicted progression or treatment outcomes; this could be due to inter-study differences in the patient population used to describe biomarkers/treatments. Future studies exploring the quality of biomarkers across patient populations and the effects of long-term therapeutic exposure in these patients could inform treatment recommendations.

## 4 Conclusion

nr-axSpA constitutes an important subgroup of axSpA with a clinical burden comparable to that of AS. This burden can be ameliorated by early and accurate diagnosis and targeted treatments. Improved education among internists on available treatment options and accelerated research to identify consistent, predictive biomarkers are needed for optimal nr-axSpA management.

## Acknowledgments

The authors acknowledge Mylene S. Serna, PharmD (UCB Pharma, Smyrna, GA) for publication coordination, and Sharon Lee, PhD and Rohini Bose, PhD (Costello Medical, Singapore) for medical writing and editorial assistance in preparing this manuscript for publication, based on the authors’ input and direction. All persons have confirmed their permission to be namely acknowledged.

## Author contributions

All authors contributed equally to the development of this publication and provided final approval for the version of the article to be published.

**Conceptualization:** Marina Magrey, Sergio Schwartzman, Natasha de Peyrecave, Victor S Sloan, Jeffrey L Stark.

**Investigation:** Marina Magrey, Sergio Schwartzman, Natasha de Peyrecave, Victor S Sloan, Jeffrey L Stark.

**Methodology:** Marina Magrey, Sergio Schwartzman, Natasha de Peyrecave, Victor S Sloan, Jeffrey L Stark.

**Resources:** Marina Magrey, Sergio Schwartzman, Natasha de Peyrecave, Victor S Sloan, Jeffrey L Stark.

**Validation:** Marina Magrey, Sergio Schwartzman, Natasha de Peyrecave, Victor S Sloan, Jeffrey L Stark.

**Visualization:** Marina Magrey, Sergio Schwartzman, Natasha de Peyrecave, Victor S Sloan, Jeffrey L Stark.

**Writing – original draft:** Marina Magrey, Sergio Schwartzman, Natasha de Peyrecave, Victor S Sloan, Jeffrey L Stark.

**Writing – review & editing:** Marina Magrey, Sergio Schwartzman, Natasha de Peyrecave, Victor S Sloan, Jeffrey L Stark.
